# PELP: Accounting for Missing Data in Neural Time Series by Periodic Estimation of Lost Packets

**DOI:** 10.3389/fnhum.2022.934063

**Published:** 2022-07-07

**Authors:** Evan M. Dastin-van Rijn, Nicole R. Provenza, Gregory S. Vogt, Michelle Avendano-Ortega, Sameer A. Sheth, Wayne K. Goodman, Matthew T. Harrison, David A. Borton

**Affiliations:** ^1^Department of Biomedical Engineering, University of Minnesota, Minneapolis, MN, United States; ^2^Department of Neurosurgery, Baylor College of Medicine, Houston, TX, United States; ^3^Department of Psychological and Brain Sciences, Texas A&M University, College Station, TX, United States; ^4^Menninger Department of Psychiatry and Behavioral Sciences, Baylor College of Medicine, Houston, TX, United States; ^5^Division of Applied Mathematics, Brown University, Providence, RI, United States; ^6^School of Engineering, Brown University, Providence, RI, United States; ^7^Carney Institute for Brain Science, Brown University, Providence, RI, United States; ^8^Center for Neurorestoration and Neurotechnology, Rehabilitation R&D Service, United States Department of Veterans Affairs, Providence, RI, United States

**Keywords:** PELP, DBS (deep brain stimulation), packet loss, LFP (local field potential), EEG

## Abstract

Recent advances in wireless data transmission technology have the potential to revolutionize clinical neuroscience. Today sensing-capable electrical stimulators, known as “bidirectional devices”, are used to acquire chronic brain activity from humans in natural environments. However, with wireless transmission come potential failures in data transmission, and not all available devices correctly account for missing data or provide precise timing for when data losses occur. Our inability to precisely reconstruct time-domain neural signals makes it difficult to apply subsequent neural signal processing techniques and analyses. Here, our goal was to accurately reconstruct time-domain neural signals impacted by data loss during wireless transmission. Towards this end, we developed a method termed Periodic Estimation of Lost Packets (PELP). PELP leverages the highly periodic nature of stimulation artifacts to precisely determine when data losses occur. Using simulated stimulation waveforms added to human EEG data, we show that PELP is robust to a range of stimulation waveforms and noise characteristics. Then, we applied PELP to local field potential (LFP) recordings collected using an implantable, bidirectional DBS platform operating at various telemetry bandwidths. By effectively accounting for the timing of missing data, PELP enables the analysis of neural time series data collected *via* wireless transmission—a prerequisite for better understanding the brain-behavior relationships underlying neurological and psychiatric disorders.

## Introduction

Targeted electrical stimulation of the brain and spinal cord has proven to be highly effective for treatment of movement disorders, mental illness, and pain (Lozano et al., 2019). However, the neural correlates of these disorders remain poorly understood. To better investigate the electrophysiological basis of these disorders and the impact of stimulation, several device manufacturers have designed “bidirectional” implants capable of concurrently stimulating and sensing from the nervous system (Stanslaski et al., 2012, 2018; Sun and Morrell, 2014; Skarpaas et al., 2019; Goyal et al., 2021). One decision point in the design of these devices is when and how to offload data. Some devices, including the Medtronic PC+S (Stanslaski et al., 2012) and NeuroPace RNS (Sun and Morrell, 2014) store data onboard the implanted hardware that can be transferred to an external computer after data collection. Other devices, including the Medtronic Summit RC+S (Stanslaski et al., 2018), the Medtronic Percept PC (Goyal et al., 2021), and the CereplexW (Yin et al., 2014; Simeral et al., 2021) enable real-time streaming of neural data to external devices meters away. These devices can be used to collect chronic neural recordings in natural environments, enabling the identification and development of personalized biomarkers and therapies (Wozny et al., 2017; Kremen et al., 2018; Gilron et al., 2021; Provenza et al., 2021).

**Figure 1:**
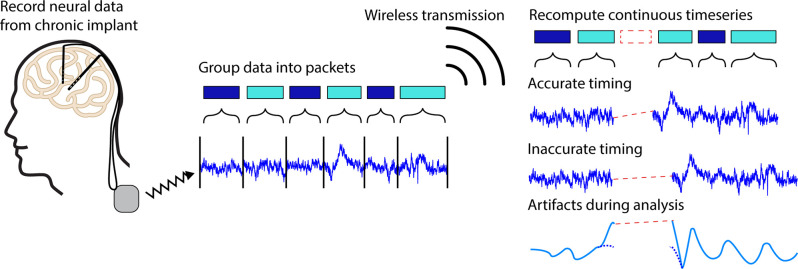
Illustration of packet loss. Neural timeseries data recorded from a chronic implant can be chronically streamed to an external receiver by grouping continuous data into discrete chunks called packets. During transmission, it is possible for packets to fail to reach the receiver leading to regions of missing data known as packet losses. In some systems, it is possible for the timing information contained in each packet to be inaccurate leading to timing offsets in neural signals of interests, artifacts during analyses, and difficulties extracting biologically relevant signals.

During wireless transmission, neural data samples are grouped into formatted units called “packets” (Bazaka and Jacob, 2012). Packets typically contain a series of subsequent samples of a particular length as well as timing information and other relevant metadata. When transmitted, it is possible for packets to fail to reach the receiver, resulting in lost packets. The timing information contained in each packet should hypothetically enable time-domain signal reconstruction. However, the metadata contained in each packet may be inexact due to hardware, network, and software delays and inaccuracies (Levesque and Tipper, 2016), resulting in uncertainty in the number and timing of missing data samples (Figure 1).

Herein we will use the Medtronic Summit RC+S as an example. In this case, a combination of inexact packet sizes and inaccurate timing variables make it difficult to exactly account for the number of lost samples (Sellers et al., 2021). Particularly in less controlled environments where the patient, telemeter, or receiver may frequently move, recordings are especially prone to packet loss (Mazzenga et al., 2002; Tsimbalo et al., 2015; Gilron et al., 2021). Lower sampling rates, and thus lower bandwidth needs, generally reduce the number of dropped packets and increase transmission ranges, however, it is still typical for as much as 5% of the data to be lost even with such adjustments. Significant work from Sellers et al. (2021) has been devoted to ensuring neural timeseries data can be accurately analyzed and aligned in the presence of packet losses. This work was designed to ensure packet timing for long-term neural recordings (>1 h) on the order of 50 ms. For short timescale effects such as individual trials in behavioral tasks, alignment errors on the order of 50 ms lead to the introduction of timing inaccuracies, artifacts during filtering, and reduced ability to identify meaningful neural signals, driving the need for more precise data reconstruction (Dastin-van Rijn et al., 2021b; Figure 1).

Here, we develop Periodic Estimation of Lost Packets (PELP) to exactly estimate packet losses in neural data collected from bidirectional, implanted devices during neurostimulation. PELP is a data-driven procedure that leverages the highly periodic and predictable nature of stimulation to accurately account for the number of samples missing due to each dropped packet. We show that PELP is robust across a range of amplitude ratios between stimulation and signal, pulse to pulse variations in stimulation amplitude, drift in stimulation frequency, and uncertainties in loss size estimates. Lastly, we successfully apply PELP to data recorded using the Summit RC+S from a human participant performing a behavioral task both in the clinic and at home to exactly estimate every occurrence of packet loss. PELP enables accurate reconstruction of the timing of missing data and facilitates analyses of neural time series data collected using bidirectional, implanted devices.

## Materials and Methods

### Periodic Estimation of Lost Packets (PELP)

**Figure 2:**
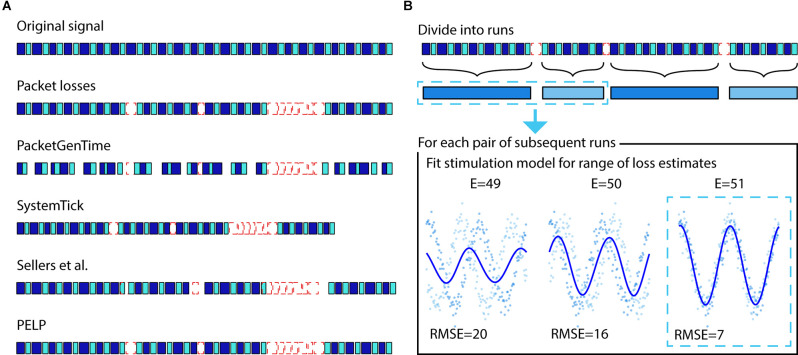
Illustration of packet loss correction and PELP. **(A)** The relative timing of the samples contained in received packets can be uncertain. Adjusting timing solely with PacketGenTime leads to many inaccurate overlaps, systemTicks will accumulate error over long recordings, the approach from Sellers et al. (2021) ensures consistency within runs but offsets at losses, while PELP can ensure exact reconstruction. **(B)** PELP begins by grouping contiguous packets (blue, first row) into continuous runs (blue, second row) where each run is separated from adjacent runs by losses (dashed-red). Loss sizes are estimated but uncertain. The stimulation period is analytically determined using all the data. For each pair of subsequent runs, the root mean squared error (RMSE) between a stimulation model and the samples in the two runs is computed for a range of loss sizes centered around the estimate (indicated by E = for each size). A new stimulation model is fit for each loss estimate. The loss size that minimizes the RMSE is selected as the true loss size.

Periodic Estimation of Lost Packets (PELP) is a method for estimating the exact number of samples missing due to a packet loss for recordings where stimulation is present. Before PELP can be applied to a recording, the locations of packet losses and their estimated sizes must first be determined. As an exemplar device, we focus our methodological development on the wireless data transmission from the Medtronic Summit RC+S; however, the methodology is generally applicable. For recordings using the Medtronic Summit RC+S, each packet has three integer timing variables of note for this purpose: “dataTypeSequence” indicating the packet number that rolls over every 256 packets, “systemTick” time of the last sample in a packet with 0.1 ms resolution that rolls over every 6.5536 s, and “timestamp” with 1 s resolution and no rollover (Sellers et al., 2021). The “dataTypeSequence” is necessary for identifying packets, “systemTick” is used for highly accurate timing over short timescales and sub-second resolution, and “timestamp” is used for highly accurate timing over long timescales and second resolution. While these specific variables are unique to the Medtronic Summit RC+S, their specific functionalities are common to wireless transmission protocols. A packet loss has occurred when the dataTypeSequence between subsequent packets skips an index or the timestamps are inconsistent with the systemTick data. For sampling rate *F*_s_ and *m* rollovers, the number of samples lost *N* can be estimated according to the following equation:


$$N=\left(\left(\left(S_{2}-S_{1}\right)\;\mathrm{mod}\;65536\right)+65536\times m\right)\times 10^{-4}-n$$

where *S*_1_ and *S*_2_ are the system ticks of the packets preceding and following the loss respectively and is the number of samples in the packet after the loss. For loss segments greater than 6 s, the resolution of a systemTick is no longer acceptably accurate due to timing drift between the systemTick and timestamp. In these cases, the timing will need to be reset using a coarser metric such as the Unix (PacketGenTime) timestamp (±50 ms vs. ±3 ms) corresponding to the time when the packet was received or generated. These estimates are not sufficiently accurate to ensure the exact reconstruction of the timing between received packets down to sample resolution. PELP leverages the presence of regular stimulation in both the received and missing data to ensure exact estimates of data losses. An illustration of the differences between timing methods is shown in Figure 2A.

Before applying PELP, we divide the time series into a set of consecutive “runs”, where each run is composed of contiguous packets, and consecutive runs are separated by packet losses. The recording in run *r* is a sequence of *n*_r_ (time, value) pairs ((*t_k,r_*,*y_k,r_*):*k* = 1,‥.,*n*_r_) where *t_k,r_* is the time relative to the start of run *r* and *y*_k,r_ is the recorded LFP amplitude at that time. Let δ be the period of stimulation, which we assume is constant across the entire recording. For regular stimulation, the stimulation artifact can be modeled as the δ-periodic function:


$$f_{\beta }\left(t\right)=\beta_{1}+\sum_{j=1}^{m}\beta_{2j}\;\sin\left(\frac{2\pi jt}{\delta }\right)+\sum_{j=1}^{m}\beta_{2j+1}\;\cos\left(\frac{2\pi jt}{\delta }\right)$$

for appropriate choice of the number of harmonics (m) and the parameter vector (*β*_j_: *j* = 1,‥.,2*m* + 1) (Dastin-van Rijn et al., 2021a). Within a single run, *β* can be estimated *via* least-squares using the harmonic regression model y_k,r_ = ƒ_β_(*t*_k,r_) + ɛ_k,r_ with homoscedastic noise ɛ_k,r_. Across multiple runs, however, the regression model will only be a good fit for appropriate choice of the packet loss sizes, a fact that we can leverage to estimate these loss sizes from data. Let Δ_r_ denote the duration of the packet loss between runs *r* and *r* + 1. We estimate Δ_r_ by choosing the one that gives the best least-squares fit to the harmonic regression model using the combined data from runs *r* and *r* + 1, i.e.,


$${\hat{\Delta }}_{r}=\overset{\arg\;\min}{\Delta \in \Omega_{r}}\overset{\min}{\beta }\left[\sum_{k=1}^{n_{r}}{\left(y_{k,r}-f_{\beta }\left(t_{k,r}\right)\right)}^{2}+\sum_{k=1}^{n_{r}+1}{\left(y_{k,r+1}-f_{\beta }\left(t_{n_{r},r}+\Delta +t_{k,r+1}\right)\right)}^{2}\right]$$

where Ω_r_ is a (small) finite set of candidate loss sizes, in our case, the set of loss sizes corresponding to a positive integer number of samples, centered around the initial loss size estimate, and spanning the uncertainty in the estimate (the range of samples we expect the true loss size to fall in). The minimization over *β* can be done exactly for each Δ ∈ Ω_r_ using least-squares, and then the optimal Δ can be selected. It is important that *Ω*_r_ is based on an accurate initial estimate without too much uncertainty because candidate loss sizes that differ by an integer multiple of the period δ cannot be distinguished. The method is illustrated in Figure 2B.

PELP requires knowledge of the stimulation period (δ) and an appropriate choice of the number of harmonics (*m*) used by the harmonic regression model. Although δ is known in principle, slight inaccuracies in device system clocks make it important in practice to use data-driven methods to estimate δ. Before using PELP, we estimate δ from combined data across all runs using the multiple-channel period estimation method described in detail in Dastin-van Rijn et al. (2021a) and Provenza et al. (2021) where we treat each run as a separate channel, where we use at most the first 10^4^ samples from each run, and where we use only the first two stages of the stagewise search for δ before the final optimization. We similarly choose in a preprocessing step using Akaike Information Criterion (AIC; Akaike, 1974) for the harmonic regression model (with Gaussian errors) applied to the single longest run.

### Participant

The research was approved by the Food and Drug Administration and conducted in accordance with the principles embodied in the Declaration of Helsinki and in accordance with local statutory requirements. The participant gave informed consent and the data presented were collected in accordance with recommendations of the federal human subjects’ regulations and under protocol H-44941/H-49125 approved by the Baylor College of Medicine Institutional Review Board. EEG data were recorded both with and without stimulation when the participant visited the clinic for DBS programming. LFP data were recorded both in the clinic and when the patient was at home. Electrodes were implanted bilaterally in the VC/VS according to standard stereotactic procedures using computed tomography for target determination. The location of electrode placement was made entirely on clinical grounds. Bilateral 150.6 Hz stimulation with a pulse width of 90 μs and amplitudes of 4 mA for the left side and 4.5 mA for the right side was used for all recordings where stimulation was turned on.

### EEG and LFP Recording Procedures

Continuous electroencephalography (EEG) was recorded using a 64-channel ActiCap BrainVision system (Brain Vision, Morrisville, NC, USA). A common mode sense electrode was located at FCz. The EEG was band-pass filtered online between 0.1 and 1,000 Hz and digitized at 5 kHz. The EEG was downsampled offline to 1,000 Hz with an anti-aliasing filter prior to analysis. The continuous LFP was recorded using the Medtronic Summit RC+S (Medtronic, Minneapolis, MN, USA) *via* wireless data streaming from implanted electrodes to the device running the task. Each DBS probe (Model 3387, one per hemisphere) contains four electrode contacts two of which were used per side to conduct bipolar recordings. LFP recordings were sampled at 1 kHz in the clinic and 250 Hz at home to minimize data losses. Signal processing and analysis were performed in MATLAB (Mathworks, Natick, MA, USA) using in-house code.

### Stimulation Simulation Procedure

**Figure 3:**
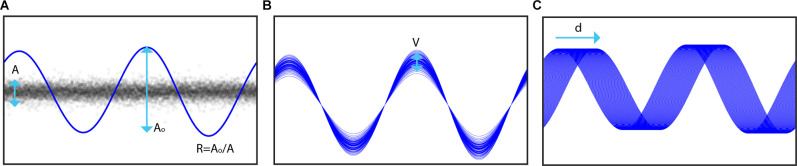
Illustration of simulation components. **(A)** The root mean squared amplitude (*A*_o_) of the stimulation model is set relative to that of the neural signal of interest (*A*) according to a target ratio *R*. **(B)** The amplitude of each stimulation pulse is varied on a cycle-wise basis where the amplitude of each pulse is sampled from a normal distribution with mean *A*_o_ and standard deviation *V*. **(C)** Inaccuracies in period estimation, drifting sampling rate, and frequency variability are modeled by adding a drift factor *d* to the stimulation period in the model.

To simulate stimulation in our recordings, we modeled DBS artifacts as a sum of sinusoidal harmonics of the stimulation frequency (Sun et al., 2014). The effect of stimulation was simulated by adding the artifact component regressed from recordings on a different day where stimulation was turned on to data without stimulation. A high-pass filter at 1 Hz with a gaussian window was first applied to achieve approximately 40 dB attenuation in the stopband before the period of stimulation (δ) was identified using the period estimation component of PARRM (Dastin-van Rijn et al., 2021a). A sum of sinusoids *ƒ*_β_(*t*) with m harmonics of the period and coefficients *β* was then fit to the data using linear regression. The stimulation amplitude for each cycle was sampled from a normal distribution with mean *A*_1_ and standard deviation *V*. The mean stimulation amplitude was set relative to the root mean squared amplitude of the stimulation off data (*A*) according to a ratio (*R*) and the original root mean squared amplitude of the fit (*A*_0_). To model potential inaccuracies in period estimation, the period of the stimulation model was slightly offset by a drift factor (*d*) measured in percent drift per 1,000 cycles from the period used during PELP. The effects of these three parameters are illustrated in Figure 3.

### Computational Experiments

We conducted three sets of experiments to simulate the accuracy of loss estimation while varying different parameters in the stimulation model. For each set, Monte Carlo analyses were used to simulate many experiments by randomly sampling subsets of 50 sample “packets” to remove from a 66 s recording. These simulations were applied while varying one of the following—amplitude ratio, amplitude variability, or drift as a function of the loss uncertainty. For each simulation, the uncertainty (for computing loss size with PELP) ranged from 0 to 50 samples in one sample increments while the dependent parameters ranged from 0 to 4 in increments of 0.1 for the amplitude ratio, 0%–10% in increments of 1% for the amplitude variability, and 0%–0.6% in increments of 0.015% per 1,000 cycles for the drift. PELP was applied to each simulation to determine the proportion of losses it was able to estimate correctly depending on the stimulation parameters. This approach is like that of Boudewyn et al. and is informative because it uses a combination of real EEG data analogous to LFP (so that the noise properties are realistic) and artificially induced losses (so the actual truth is known) across a range of modeled stimulation waveforms (Boudewyn et al., 2018).

## Results

### Stimulation Model Fit

**Figure 4:**
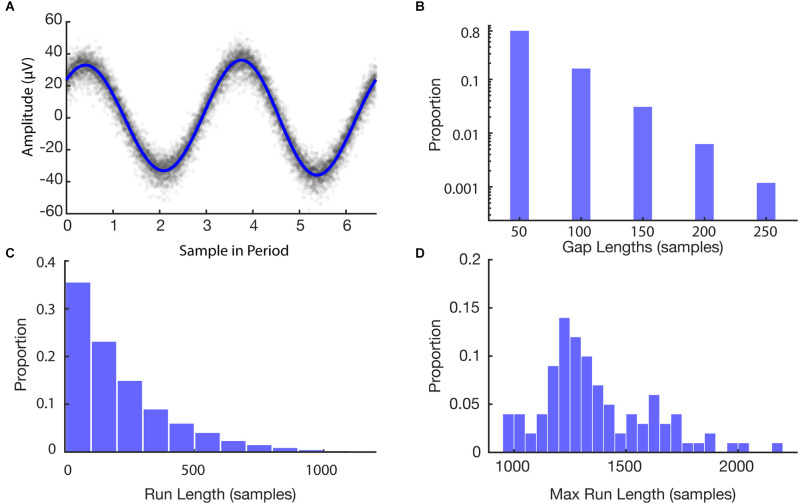
Features of simulation. **(A)** Stimulation model fit to EEG data. Raw data are shown in gray and the model is shown in blue. **(B)** Histogram of missing data gap lengths for all experiments. **(C)** Histogram of continuous run lengths for all experiments. **(D)** Histogram of longest continuous runs in each experiment.

We first sought to estimate the frequency of stimulation to construct a model for time series data alignment. Figure 4A shows the stimulation model compared to the raw EEG samples overlapped by computing the modulus of each timepoint with the model’s period of stimulation. The period of stimulation was found to be 6.64000 samples. Four sinusoidal harmonics were used for the fit based on model selection *via* AIC. Raw samples are well consolidated about the artifact waveform with a residual standard deviation of 5.90 μV similar to the standard deviation of the stimulation off data (4.55 μV).

### Loss Simulations

After building the model of stimulation times, we then ran a set of simulations to provide bounds on the expected recovery and overall loss after using the PELP method for data alignment. The histograms in Figure 4 illustrate features of the Monte Carlo simulation of 100 loss experiments. The average length of a missing data gap was 63 samples with a median of 50 samples (one loss; Figure 4B). Runs of continuous samples between losses ranged from 50 to 2,200 samples with an average length of 251 samples (Figure 4C). The max run length in each simulation ranged from 950 to 2,200 samples with an average length of 1,342 samples corresponding to roughly 202 cycles of the 150.6 Hz simulated stimulation frequency (Figure 4D).

### Loss Estimation Experiments

**Figure 5:**
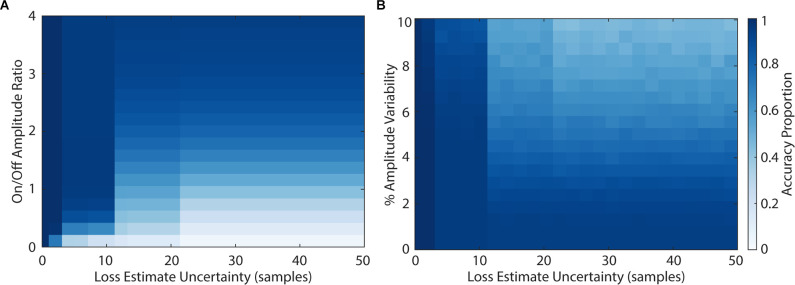
Accuracy of loss estimation as a function of amplitude ratio, amplitude variability, and uncertainty. The accuracy of loss estimation was computed for 100 simulated trials with 20% of the packets removed. More accurate parameter combinations are indicated by darker values in the colormap. Amplitude ratios **(A)** ranged from 0 to 4, amplitude variability **(B)** ranged from 0% to 10%, and uncertainty ranged from 0 to 50 samples.

We then explored the impact of model parameters on the loss of sensing data using PELP. Figure 5 shows the Monte Carlo simulated loss experiments measuring the accuracy of PELP estimates as a function of the stimulation amplitude ratio, amplitude variability, and estimate uncertainty. Both heat maps show discrete transitions in accuracy at uncertainties of 3, 6, 11, and 21 samples. This occurs because estimate differences at these multiples are more closely overlapping than others for the specific stimulation period of 6.64 samples. The magnitude of estimation errors also increased at these transitions with most errors corresponding to 1, 3, 6, 11, or 21 samples of offset depending on the uncertainty. For constant uncertainty, accuracy increased smoothly for increasing amplitude ratio and decreasing amplitude variability. Changes in drift, in the range tested, had no effect on accuracy. Keeping amplitude ratio or amplitude variability constant while varying uncertainty had little effect on accuracy with exception of the effects at multiples of three samples. Changes in uncertainty for constant drift had no effect on accuracy. Accuracy was near 100% for amplitude ratios above 0.2 for uncertainties less than three samples, amplitude ratios above 0.5 for uncertainties less than nine samples, and amplitude ratios above three for uncertainties greater than nine samples. Accuracy was near 100% for amplitude variabilities below 2% for uncertainties less than nine samples. For uncertainties larger than nine, amplitude variability had to be near zero to maintain 100% estimate accuracy. Across all values for the drift experiment, accuracy was near 100%.

### PELP With Medtronic Summit RC+S Recordings

**Figure 6:**
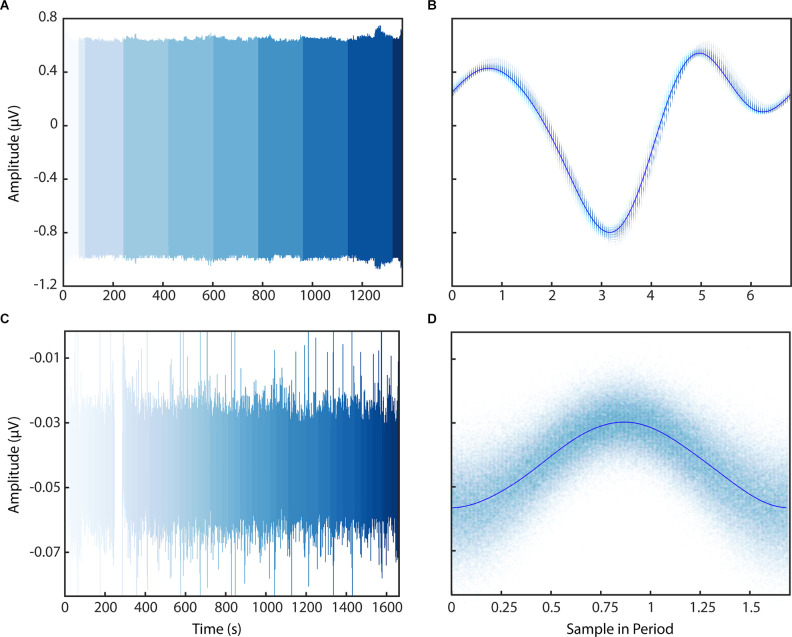
Application of PELP to data from the Medtronic Summit RC+S. PELP was applied to RC+S data containing losses sampled at 1,000 Hz **(A,B)** and 250 Hz **(C,D)**. Each continuous run is indicated by a distinct shade in the colormap **(A–C)**. For both conditions, samples from all runs were overlapped on the timescale of the period of stimulation **(B–D)**. Samples were well consolidated in the stimulation model for both conditions indicating accurate estimation of loss sizes.

We then applied the PELP methodology of offline data realignment to brain recordings collected from participants of an ongoing clinical study to demonstrate real world performance. Figure 6 shows LFP data from a behavioral task containing packet losses recorded using the Medtronic Summit RC+S in the clinic and at home after the estimation of losses using PELP. Data recorded in the clinic sampled at 1,000 Hz contained 15 losses with a median size of 200 samples (Figures 6A,B). Data recorded at home sampled at 250 Hz contained 121 losses with a median size of 17 samples (Figures 6C,D). When overlapped on the timescale of the period of stimulation, all samples from both conditions were well consolidated about the stimulation waveform with no observable evidence of significant period drift indicating that losses were accurately accounted for. In contrast to PELP, if the original loss size estimates (Supplementary Figure 1A) or the method from Sellers et al. (2021; Supplementary Figure 1B) are used for loss size estimation, the samples are not consolidated about the stimulation waveform indicating that losses are not accurately accounted for.

## Discussion

Streaming of intracranial electrophysiology data in ecologically valid environments is essential for biomarker discovery in a variety of neurological disorders. Bidirectional implanted devices have enabled the acquisition of such datasets, however, data losses during wireless streaming hinder accurate analyses of neural signals. To address these challenges, we have developed PELP to exactly estimate and account for data losses from implanted recordings where stimulation is on. We show using simulations of data losses that PELP can accurately estimate missing samples over a variety of stimulation conditions. Lastly, we successfully applied PELP to reconstruct the timing of data recorded using the Medtronic Summit RC+S using various telemetry settings amenable for both stationary and ambulatory streaming in the laboratory and in natural environments.

PELP is applicable for precise packet alignment for the Summit RC+S and may be applicable to other stimulating devices capable of wireless data streaming with similar acquisition protocols. Our stimulation model accurately accounts for the range of amplitude and variability parameters that could be expected for other implanted devices. In recordings where sensing and stimulation occur on nearby contacts, stimulation amplitude can exceed the underlying neural signal by a factor of 10 (Allen et al., 2010). For recordings where sensing and stimulation occur far apart or the stimulation harmonics fall within the transition band of an online low-pass filter, the amplitude ratio will be closer to 1. Our recordings using the Summit RC+S had amplitude ratios of 27 and 1.2 and estimate accuracy was consistent with predictions from the simulation (as determined from visual inspection of the consolidated stimulation artifact). Pulse to pulse amplitude variability for the Summit RC+S is well within the range of values where PELP was most accurate. Fluctuations in the battery or the surrounding medium could influence amplitude on longer timescales. While only the run nearest to the loss was used for estimation, drift within the run itself would not be well accounted for. Exceptionally long runs or runs where the drift was identified could be divided to improve estimation accuracy. Similar considerations would also be effective if stimulation frequency drift or errors in period estimation occur.

In the case of clinical devices, it is necessary to work within the confines of the hardware available with little ability to inject external sources of information. In research contexts, where experimental design and data acquisition are more flexible, data losses are handled by utilizing timing systems with greater accuracy and minimal rollover or the injection of reference signals into the recording system that ensure timing is unambiguous. Neither of these solutions is feasible for a fully implanted, closed-source, clinical system necessitating a solution like PELP.

Since PELP requires stimulation artifacts to be present to model the signal during data losses, the method is not applicable for recordings where stimulation is off or significantly attenuated by online filters. In such circumstances, less accurate methods utilizing packet timing metadata must be used for loss estimation. In theory, stimulation could be applied below therapeutic amplitudes and still be used for reconstruction using PELP. However, such modifications would only be reasonable if the inevitable stimulation artifacts did not obscure neural signals of interest. Additionally, the version of PELP described in this manuscript would not be applicable for data streamed during time-varying stimulation (closed-loop DBS). PELP could still be applied if the exact stimulation period and parameters are known for the data neighboring each loss allowing for accurate loss estimation despite changing parameters.

While PELP is highly effective for correcting inaccuracies in loss sizes, the best practice for future devices would certainly be to avoid collecting ambiguous datasets *via* synchronization approaches with consistently accurate clocks and constant packet sizes. We intend PELP to be used as a *post hoc* remediation step for existing datasets where such proactive measures cannot be made. With the Summit RC+S alone, there are several published studies where PELP could be useful for *post hoc* data cleaning (Kremen et al., 2018; O’Day et al., 2020; Petrucci et al., 2020; Gilron et al., 2021; Gregg et al., 2021; Johnson et al., 2021; Provenza et al., 2021). PELP improves over existing solutions (Sellers et al., 2021) for analyses requiring highly accurate timing for small loss sizes, thereby reducing timing offsets, delocalization, and attenuation (Dastin-van Rijn et al., 2021b). In these circumstances, PELP enables near perfect timing and could enable biomarker exploration and task-locked analyses for these studies.

## Data Availability Statement

A sample dataset and a MATLAB implementation of PELP are available at: https://github.com/neuromotion/PELP, further inquiries can be directed to the corresponding author.

## Ethics Statement

The studies involving human participants were reviewed and approved by Baylor College of Medicine Institutional Review Board. The patients/participants provided their written informed consent to participate in this study.

## Author Contributions

ED, NP, MH, and DB contributed to the conception and design of the method. ED completed all analyses and figures with input from NP, MH, and DB. NP, WG, SS, GV, and MA-O conducted LFP recordings with the participant. ED wrote the first draft of the manuscript. NP, MH, and DB wrote sections of the manuscript. WG and SS performed the clinical care aspects of the study. SS performed the study surgical procedures. All authors contributed to the article and approved the submitted version.

## Conflict of Interest

Medtronic Summit RC+S devices were provided for this study to DB and WG without charge by Medtronic as part of the NIH BRAIN public-private partnership. A provisional patent application has been filed by Brown University on behalf of ED, NP, MH, and DB on PELP. SS is a consultant for Boston Scientific, Zimmer Biomet, and Neuropace. The remaining authors declare that the research was conducted in the absence of any commercial or financial relationships that could be construed as a potential conflict of interest. The handling editor JW declared a past co-authorship with the author SS.

## Publisher’s Note

All claims expressed in this article are solely those of the authors and do not necessarily represent those of their affiliated organizations, or those of the publisher, the editors and the reviewers. Any product that may be evaluated in this article, or claim that may be made by its manufacturer, is not guaranteed or endorsed by the publisher.
