# Supplementary material for: PELP: Accounting for Missing Data in Neural Time Series by Periodic Estimation of Lost Packets
Source: Front Hum Neurosci. 2022 Jul 7;16:934063. doi: 10.3389/fnhum.2022.934063 (PMC9301255; doi:10.3389/fnhum.2022.934063)
Supplement: Supplementary file 1 [file Data_Sheet_1.DOCX]

Supplementary Material


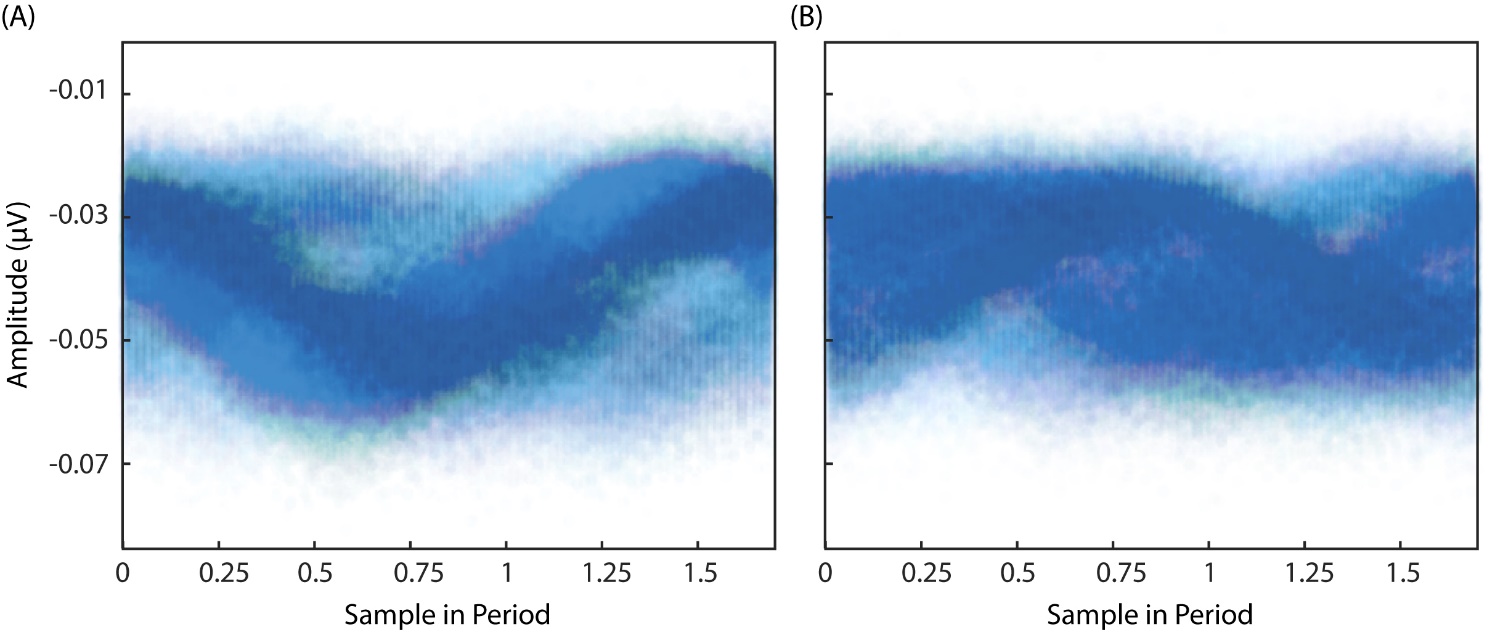


**Supplementary Figure 1.** **Sample overlap for alternatives to PELP.** Samples from all runs were overlapped on the timescale of the period of stimulation using the original loss size estimates **(A)** and the method from Sellers et al. **(B)**. Samples were not well consolidated for either condition indicating inaccurate estimation of loss sizes.
